# A computational grid-to-place-cell transformation model indicates a synaptic driver of place cell impairment in early-stage Alzheimer’s Disease

**DOI:** 10.1371/journal.pcbi.1009115

**Published:** 2021-06-16

**Authors:** Natalie Ness, Simon R. Schultz

**Affiliations:** Centre for Neurotechnology and Department of Bioengineering, Imperial College London, London, United Kingdom; École Normale Supérieure, College de France, CNRS, FRANCE

## Abstract

Alzheimer’s Disease (AD) is characterized by progressive neurodegeneration and cognitive impairment. Synaptic dysfunction is an established early symptom, which correlates strongly with cognitive decline, and is hypothesised to mediate the diverse neuronal network abnormalities observed in AD. However, how synaptic dysfunction contributes to network pathology and cognitive impairment in AD remains elusive. Here, we present a grid-cell-to-place-cell transformation model of long-term CA1 place cell dynamics to interrogate the effect of synaptic loss on network function and environmental representation. Synapse loss modelled after experimental observations in the APP/PS1 mouse model was found to induce firing rate alterations and place cell abnormalities that have previously been observed in AD mouse models, including enlarged place fields and lower across-session stability of place fields. Our results support the hypothesis that synaptic dysfunction underlies cognitive deficits, and demonstrate how impaired environmental representation may arise in the early stages of AD. We further propose that dysfunction of excitatory and inhibitory inputs to CA1 pyramidal cells may cause distinct impairments in place cell function, namely reduced stability and place map resolution.

## Author summary

Cognitive decline in Alzheimer’s Disease (AD) correlates most strongly with dysfunction and loss of synapses in affected brain regions. While synaptic dysfunction is a well-established early symptom of AD, how impaired synaptic transmission may lead to progressive cognitive decline, remains subject to active research. In this study, we examine the effect of synapse loss on neuronal network function using a computational model of place cells in the hippocampal network. Place cells encode a cognitive map of an animal’s environment, enabling navigation and spatial memory. This provides a useful indicator of cognitive function, as place cell function is well characterized and abnormalities in place cell firing have been shown to underlie navigational deficits in rodents. We find that synapse loss in our network is sufficient to produce progressive impairments in place cell function, which resemble those observed in mouse models of the disease, supporting the hypothesis that synaptic dysfunction may underlie the cognitive impairment in AD. Furthermore, we observe that loss of excitatory and inhibitory synapses produce distinct spatial impairments. Future experiments investigating the relative contribution of different synaptic inputs may thus allow new insights into the neuronal network alterations in AD and potentially enable the identification of new therapeutic targets.

## Introduction

AD is the most common cause of dementia, marked by progressive memory impairment and other cognitive deficits that ultimately lead to a loss of functional independence [1]. The neurodegeneration in AD is associated with a reduction in neuronal activity and neuronal atrophy [1–3]. However, in patients and mouse models of AD, cognitive impairment correlates most strongly with synaptic dysfunction, which precedes cellular loss, suggesting AD is primarily a synaptopathology [4–8]. Furthermore, pathological forms of amyloid *β* and tau protein, key hallmarks of AD, have been found to trigger numerous molecular cascades that induce synaptic dysfunction and loss [4, 9, 10].

While it is well established that AD reduces neuronal activity, clinical observations, including the occurrence of epileptic seizures, suggest a more complex derangement of activity [11]. Recent neuroimaging studies have revealed that hyperactivity is the primary neuronal dysfunction in early AD, and may further exacerbate disease progression by increasing the release of amyloid *β* and tau protein [12–14]. There is substantial evidence suggesting an underlying disturbance of the excitation-inhibition balance as a result of impaired synaptic transmission [15]. For instance, impairments in slow-wave propagation in an APP/PS1 mouse model could be rescued by enhancing GABAergic inhibition [16]. An enhanced understanding of neuronal network alterations and the underlying actors may enable the identification of new therapeutic targets, and subtle changes in hippocampal activity provide promising early and easily-accessible biomarkers for diagnosis [17–19].

Pathological lesions of AD are primarily found in the hippocampus and associated structures [1]. Correspondingly, declarative episodic memory and navigation, which involve processing in the hippocampus, are typically impaired early [20]. Navigation and spatial memory require the activity of place cells, which encode a ‘cognitive map’ of an animal’s environment [21], and spatial impairments correlate with abnormalities in place cell activity in mouse models of AD [22–25]. Further experiments examining place cell dysfunction in early AD may shed light on how hippocampal network dysregulation is related to cognitive deficits. However, this is a challenging question to address experimentally, as it requires longitudinal tracking of individual cells and synaptic transmission. Using optical imaging, registration of the same neurons becomes increasingly difficult as the number of and interval between sessions increase [26]. Computational modelling may enable identification of critical timepoints and functional links to refine such *in vivo* experiments. The aim of this study therefore was to create a functional model of long-term place cell dynamics in hippocampal region CA1, in order to investigate how AD-related changes in connectivity impair network function throughout disease progression.

The computational model we have used here is based on a feedforward grid-cell-to-place-cell transformation, which has previously been used with Hebbian learning and competitive network interactions to produce place field formation [27, 28]. Models of grid-cell-to-place-cell transformations generally use the summed input from grid cells to hippocampal cells together with competitive network interactions to obtain highly spatially selective activity [27, 29–32]. A notable exception is the spike-based model by Savelli and Knierim [33], in which Hebbian plasticity alone in the absence of competitive network interactions accomplished place field formation in single cells, suggesting that the role of network interactions may primarily be in the coordination of firing fields across an environment. Interestingly, De Almeida et al. [27] showed that given a sufficiently vast range of initial synaptic weights, competitive network interactions between cells can generate place fields without the need for synaptic plasticity. Acker et al. [28] have expanded this model to account for long-term persistence of place fields in CA1 despite the recently established mean synapse lifetime of only 10 days in CA1 [34, 35]. They incorporated daily synaptic turnover in line with experimental findings in CA1 and demonstrated that Hebbian learning is sufficient to retain stable place fields over multiple weeks in the presence of frequent synaptic restructuring. Their model suggests that memory can persist in the correlation structure of a dynamic network with synaptic plasticity, thereby outliving individual synapses (for further reading, see review by [36]). The model presented by Acker et al. [28] specifically shows that after some synapses are turned over, the remaining synapses will influence the learning of newly formed synapses, such that activity within the firing field of the cell will be maintained. This allowed for memory to be retained even when all original synapses have been turned over, suggesting that synaptic restructuring is compatible with long-term memory within a network. Our model incorporated the competitive network interactions presented by De Almeida et al. [27] and the synaptic turnover and plasticity from the Acker et al. [28] model to obtain long-term persistent place fields from grid cell inputs.

There is substantial evidence supporting the biological plausibility of grid cells as the major determinant of place cell firing [37]. Grid cells are not only the most abundant type of spatially-modulated cell in the superficial entorhinal cortex (EC) [38], but also comprise a major proportion of the excitatory input to place cells [39]. In addition, place field size decreases in response to EC lesions [40], grid cell realignment coincides with place cell remapping [41] and grid cells have been implicated in the temporal organisation of place cell firing [42, 43]. In the absence of other inputs, direct EC input is sufficient to establish and maintain place cell firing [44], although spatial information content is reduced [45].

The model we present here is to our knowledge the first grid-cell-to-place-cell transformation model able to recapitulate the persistent place fields and stable place cell density of CA1 place cell behaviour over long timescales [46–50]. Our modeling results provide strong support for the hypothesis that synaptic dysfunction drives cognitive impairment in early AD by disturbing the firing homeostasis of cortico-hippocampal circuits [15, 51, 52]. We further predict that excitatory and inhibitory synaptic dysfunction have distinct effects on place cell function in AD. In addition to opposing effects on mean firing rate, inhibitory synapse loss resulted in a lower resolution place map, while excitatory synapse loss reduced the stability of place maps over time, suggesting a direction for future experimental work.

## Results

To study the effect of AD-related synapse loss on hippocampal place coding, we simulated a population of CA1 pyramidal cells, which received excitatory input from grid cells and inhibitory feedback from interneurons, beginning from the model of Acker et al. [28]. Place cell function was analysed in a 1 m linear environment, with cells characterised by their firing rates within each 1 cm bin along the track. The model was constrained to parameters that reproduced key characteristics of long-term CA1 place coding (see Methods). We then analysed and contrasted the effect of progressive excitatory and inhibitory synapse loss on network function to examine whether AD-related synapse loss may induce impairments in place cell function.

### Key requirements for long-term stability of CA1 place cell dynamics

Our implementation of the model presented by Acker et al. [28], here referred to as ‘model 1’, involved 10,000 grid cells and 2,000 pyramidal cells and produced dense place fields. We ran multiple iterations of the simulation to investigate its properties over time, with each iteration involving synaptic turnover, competitive feedback inhibition and one session of Hebbian learning in the familiar enclosure (see Methods section for more details). As described by Acker et al. [28], the magnitude of synaptic turnover in this model is based on the extent of synaptic turnover observed experimentally in CA1, such that one iteration of the model roughly represents the structural remodelling that occurs during a day in the animal. In later versions of the model we also incorporate further experimental data on synaptic loss, which is fitted based on real calendar days. We thus refer to each iteration of the model as a ‘day’, however it is important to keep in mind that this is a simplification and does not specifically refer to a calendar day for an animal. There are many processes other than synaptic turnover that occur during a normal day of an animal and may affect spatial memory, such as modifications during sleep, and the rate of learning used here may not represent the amount of learning achieved by an animal during a day. Thus, a ‘day’ in our simulation more specifically refers to elapsed time in terms of the number of iterations that have been carried out.

**Fig 1:**
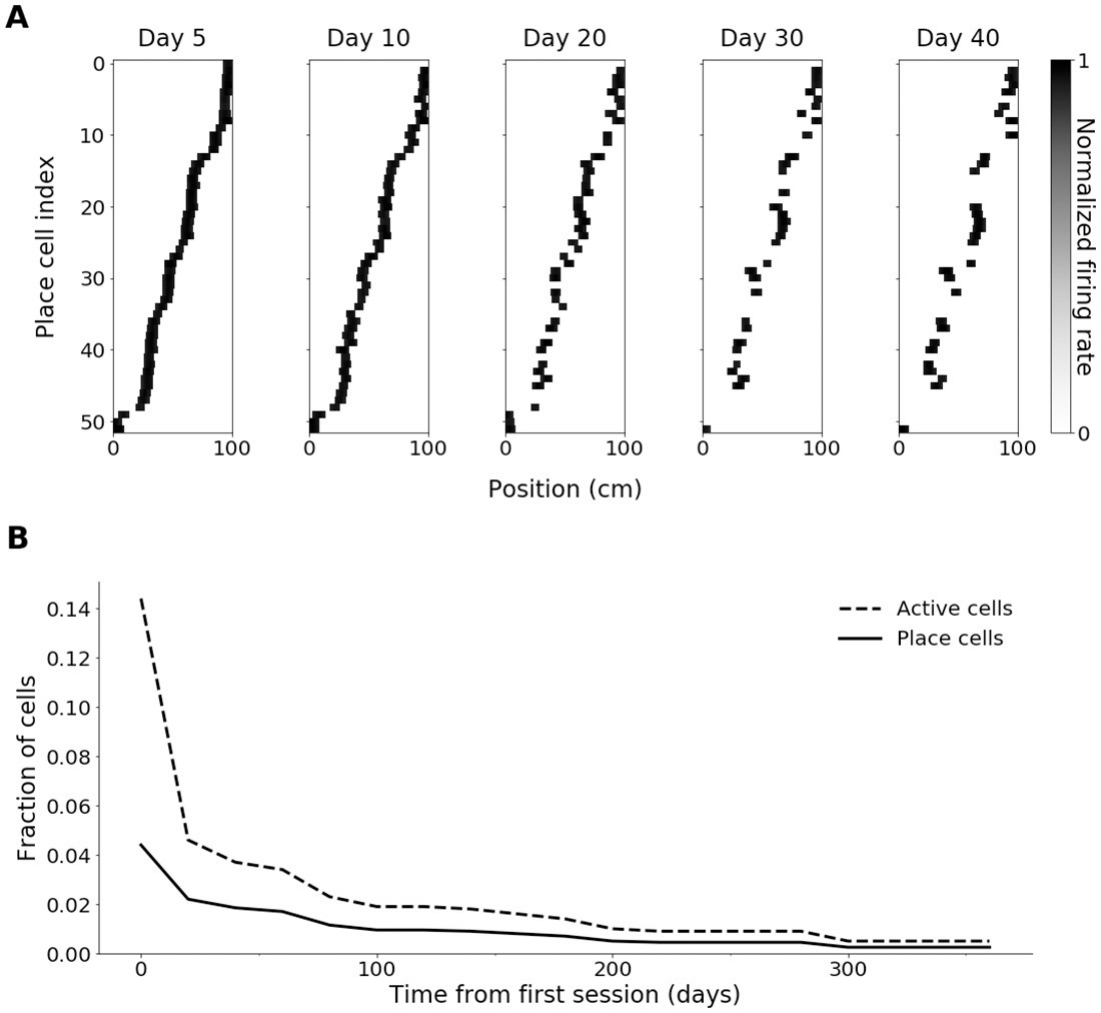
Simulation with long-term stability of place fields without novel place field formation. (A) Place field maps of cells with significant place fields on day 5, for days 5 to 40, indexed according to centroid position along the track on day 5. (B) Proportion of active cells and place cells over 365 days. Simulations were carried out with Hebbian learning. The results of a single run of the simulation of model 1 are shown.

In model 1, cells were observed to lose their place fields or remap over time, while a subset of cells retained stable place fields (Fig 1A), as observed experimentally [49, 50]. However, the number of cells with significant place fields declined rapidly due to a lack of novel place field formation (Fig 1B), leaving only the subset of ‘long-term stable’ place cells. We therefore further constrained the model to those parameters that reproduced the stable place cell density observed experimentally [50, 53], which involved shorter feedback inhibition cycles, additional homeostatic constraints and a more realistic network architecture.

### Improved feedback inhibition and synaptic plasticity

In model 1, interneuron-mediated feedback was approximated as an algorithmic global feedback mechanism without modelling individual interneurons and involved inhibition of pyramidal cells with firing rates below a given fraction of the highest firing rate. As interneurons are known to generally provide local inhibition through feedforward and feedback projections, with relatively few cases of inhibitory projections outside their local area [54], we implemented a local feedback inhibition algorithm in which interneurons provide inhibition for individual bins along the track. This promoted firing in positions of low general activity with otherwise low place field coverage.

While synaptic scaling kept the average synaptic weight constant, the proportion of active cells decreased over time (Fig 1B). Strategies that aimed to counteract this instability by reducing competition included lower levels of Hebbian plasticity and constraints on synaptic weights and weight change, however, these approaches diminished stability, causing strongly fluctuating place cell numbers with no long-term stable cells.

To introduce additional homeostatic constraints, Hebbian learning was replaced by the Bienenstock-Cooper-Munro (BCM) learning rule [55]. BCM learning relies on basic Hebbian principles but incorporates a dynamic threshold for weight change that depends on recent postsynaptic activity and enables bidirectional modification of synapse strength. Consequently, high firing rates increase the threshold for potentiation and may result in synaptic depression instead, thus increasing competition. We refer to this version of the model with local feedback inhibition and BCM learning as ‘model 2’. Under BCM learning, the rate of novel place field formation increased slightly in comparison to model 1, but not sufficiently, to balance place field loss. Additional unsuccessful approaches aimed to stabilize place field density, which were not retained in the final model, are described in S1 Text.

### Stable place cell density through improved connectivity

**Fig 2:**
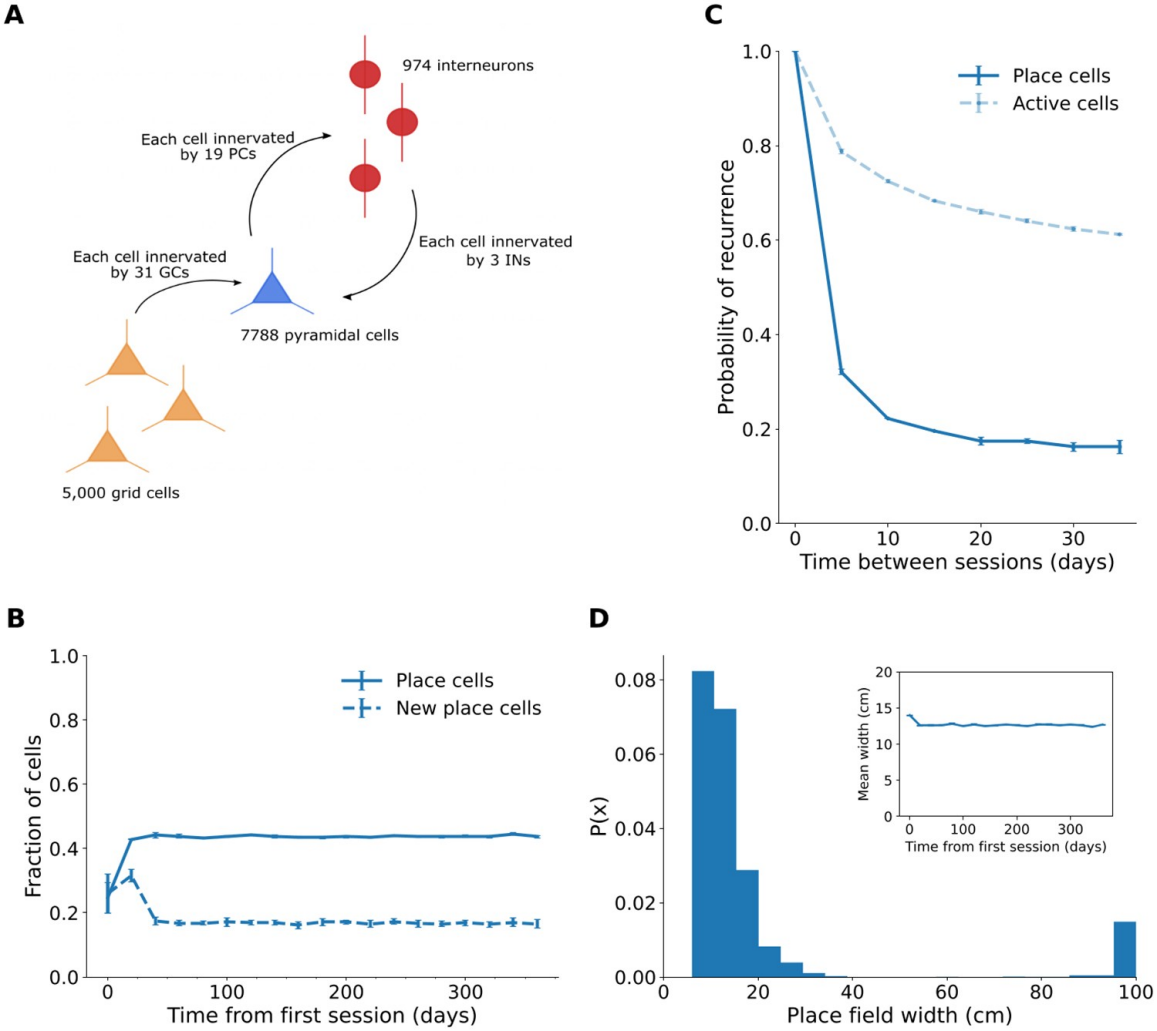
Stable place cell density and properties in the scaled model. (A) Schematic of new CA1 connectivity in model 3. (B) Proportion of total and new place cells over 365 days. (C) Probability of recurrence for active cells and place cells across sessions 5 to 35 days apart. Error bars in (B) and (C) show standard deviation across two runs of the simulation. (D) Representative probability density function of place field width. Inset: Mean place field width over 365 days, excluding widths > 50 cm. Error bars show pooled standard error of the mean across all place fields in 2 runs of the simulation. Simulations were carried out with BCM learning.

In our final version of the model, model 3, the CA1 network was scaled according to relative cell and synapse numbers in the rat [56](Fig 2A), as there was less extensive data available for the mouse. Layer III of the EC is estimated to consist of 250,000 principal cells, 80% of which are spatially tuned [57, 58]. Using estimated CA1 population sizes [56], this gives a pyramidal to grid cell ratio of 1.5575:1 and a pyramidal cell to interneuron ratio of 8:1. Due to the vast diversity and incomplete functional characterisation of interneurons, a single hypothetical type of ‘average’ interneuron was used. For simplicity, recurrent synaptic connections among pyramidal cells and among interneurons were omitted.

As a result of lower excitatory synaptic density, pyramidal cell firing rates were lower than in model 1 and 2 but remained stable. The mean firing rate over 365 days was 0.52 Hz, with a range of 0–8.23 Hz, which corresponds closely to mean firing rates detected previously in CA1 place cells *in vivo* [59–62]. Interestingly, the network reproduced the stable place cell density observed experimentally, with cells dynamically losing and gaining place fields (Fig 2B and 2C). On average, 61% of cells were active each day, 72% of which formed significant place fields. Place field width ranged between 5 − 40 cm with a mean width of 12.6 cm (Fig 2D). As some cells fired throughout most of the track but there were no place fields of intermediate width (Fig 2D), cells with firing fields of > 50 cm were not classified as place cells.

Stable place field emergence in this model suggests the high synaptic density in the previous models may have been a limiting factor by restricting variability in cell input even in the presence of high synaptic turnover.

The probability of recurrence of place cells with place fields that have drifted less than 5 cm between any two sessions, a measure of how likely it is for a cell to retain its place field, decreased from 98% and 51.9% for sessions 5 and 30 days apart, respectively, in model 1, to 32% and 17.2% in model 3 (Fig 2B). At stable place cell density, this indicates increased variability in the place cell ensemble.

At sufficient network size, determined to be approximately 5,000 grid cells and 7,788 pyramidal cells, the general features of model 3 are robust to cell number changes. However, as the model is scaled up, competition increases, leading to a lower proportion of place cells (10,000 grid cells: mean ± SEM = 19.9% ± 0.2%; 7,500 grid cells: 28.5% ± 0.1%; 5,000 grid cells: 43.8% ± 0.1%; one-way ANOVA test *F*(2,18) = 7164.82, *p* < 7.6^−27^; *N* = 18 time points from a single run of the simulation per condition).

The behaviour of the model, including stable place cell density and constant mean place field width, is also robust to varying rates of synaptic turnover (S1(B) and S1(C) Fig), although place cell stability can be tuned by the synaptic turnover rate (S1(A) Fig).

The general features are also robust to the choice of inhibition model, although using an ‘E%-max’ inhibition model, in which cells whose firing rate is within 10% of the most excited cell escape inhibition, as opposed to a ‘winner-takes-all’ model, increases the proportion of place cells due to lower competition (winner-takes-all: mean ± SEM = 29.0% ± 0.2%; E%-max: 43.8% ± 0.1%; *N* = 18 time points from a single run of the simulation per condition). The ‘E%-max’ model was used here as it accounts for feedback delay [63].

### Excitatory synapse loss reduced firing and place map stability

In APP/PS1 mouse models, progressive reductions in spine density on the distal dendrites of CA1 pyramidal neurons, which are innervated by EC layer III, have been detected at 4–15 months of age [64], with a similar extent of loss occurring in 5xFAD models [65].

**Fig 3:**
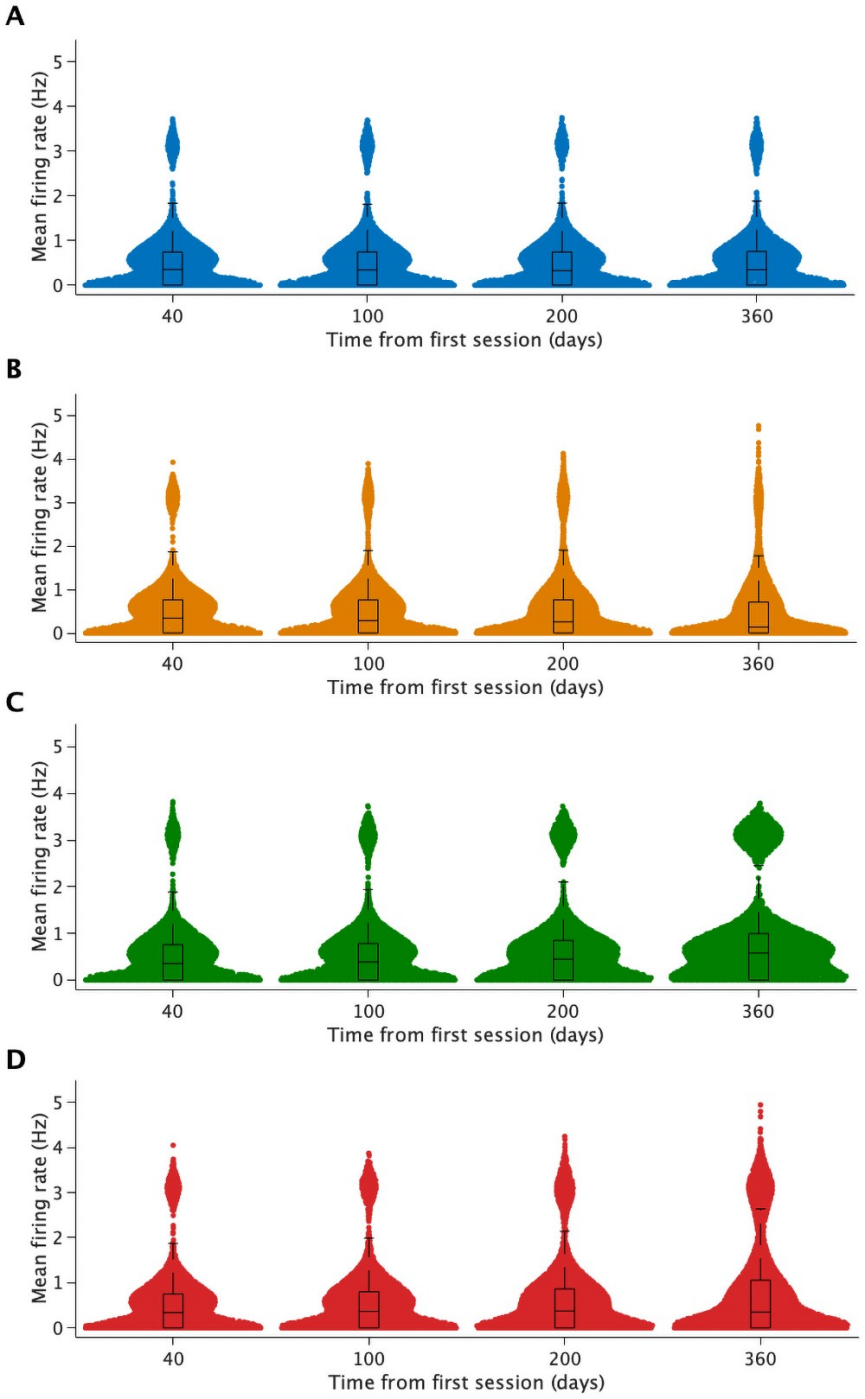
Each model exhibits characteristic changes in firing rate. Swarm plots showing mean firing rates of CA1 pyramidal cells from day 40 to day 360 in the wildtype (A), the grid-to-pyramidal-cell synapse loss model (B), the interneuron-to-pyramidal-cell synapse loss model (C) and the double synapse loss model (D). Boxplots show median and interquartile range. The results of a single run of each simulation are shown. Histograms of the same data are shown in S2 Fig.

This extent of grid-to-pyramidal cell synapse loss in our model gradually reduced the median firing rate over time, while increasing the range of firing rates (day 40: median ± IQR = 0.34 ± 0.76 Hz, range = 3.9 Hz; day 360: 0.13 ± 0.71 Hz, range = 4.8 Hz). In addition, the distribution of firing rates gradually became more uniform with less strongly pronounced ‘bumps’ (Fig 3A and 3B, S2(A) and S2(B) Fig).

**Fig 4:**
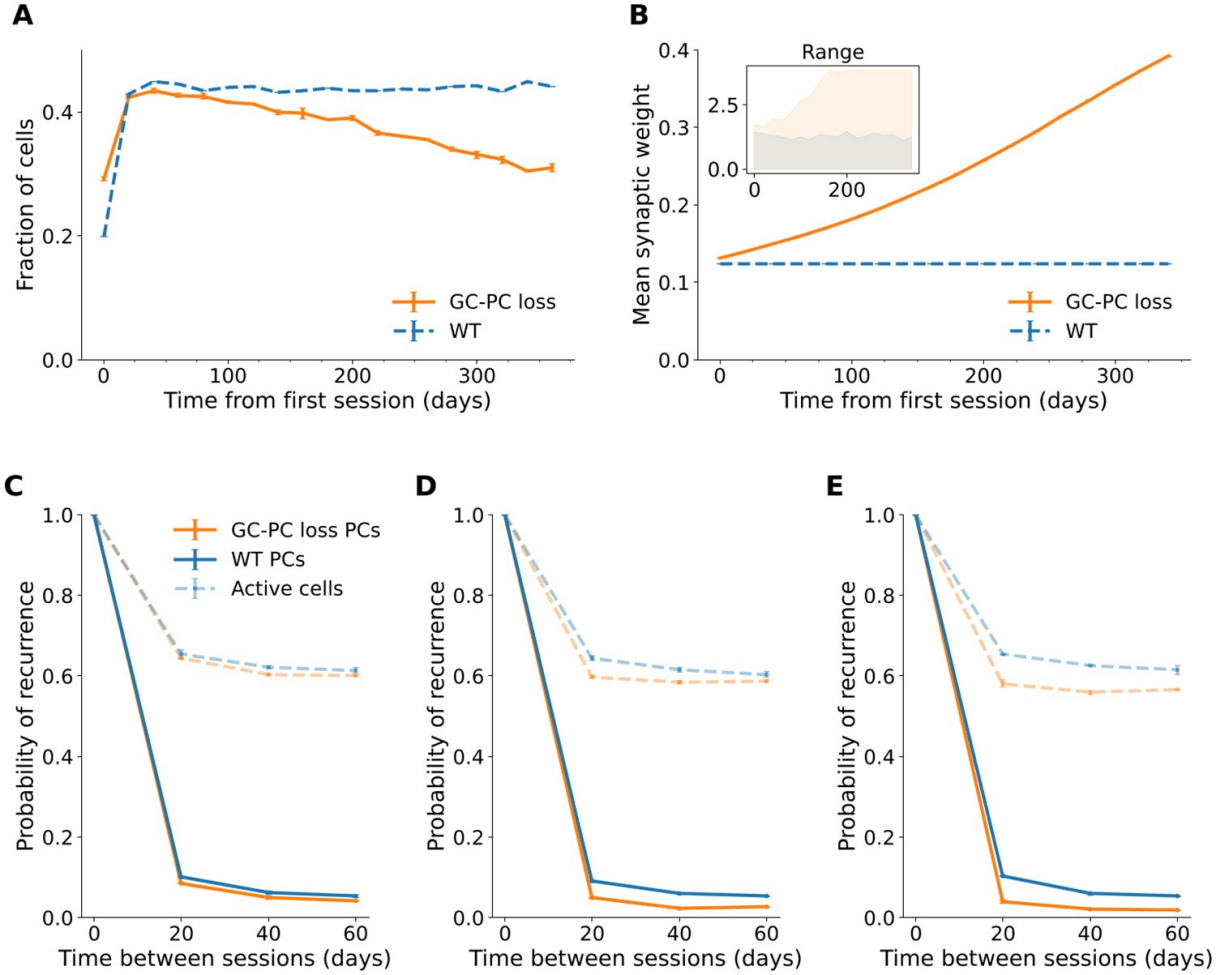
Place cell proportion and place map stability decreased as excitatory synapses were lost. (A) Proportion of place cells over 365 days in the grid-to-pyramidal-cell (GC-PC) synapse loss and wildtype (WT) model. Error bars show standard deviation across two runs of the simulation. (B) Mean synaptic weight over 365 days. Error bars show pooled standard error of all synaptic weights over two runs of the simulation. Inset: Range of weights in the GC-PC loss model (orange) and the wildtype (blue). (C-E) Probability of recurrence for place cells and active cells across sessions 20 to 60 days apart from day 20 (C), day 200 (D) and day 300 (E). Error bars show standard deviation across two runs of the simulation.

Synaptic scaling led to a compensatory increase in the mean and range of synaptic weights (Fig 4B), which likely mediated the relatively small reduction and increased variability of mean firing rates over time.

The proportion of place cells decreased over time with excitatory synapse loss (Fig 4A), while the remaining cells retained a constant place field width (mean ± SEM = 12.6 ± 0.03 cm, *N* = 49,828 place fields from 18 time points). The reduced number of place cells may have slightly diminished coverage of the environment. In addition, the probability of recurrence of active cells and place cells between sessions 20 days apart, declined over time (Fig 4C and 4E), indicating lower stability due to excitatory synapse loss.

### Inhibitory synapse loss increased neuronal activity and place field size

Progressive axonal loss, with normal bouton density on unaffected axons, has been detected on CA1 O-LM interneurons in 4–11 months old APP/PS1 mice in two separate studies [66, 67].

**Fig 5:**
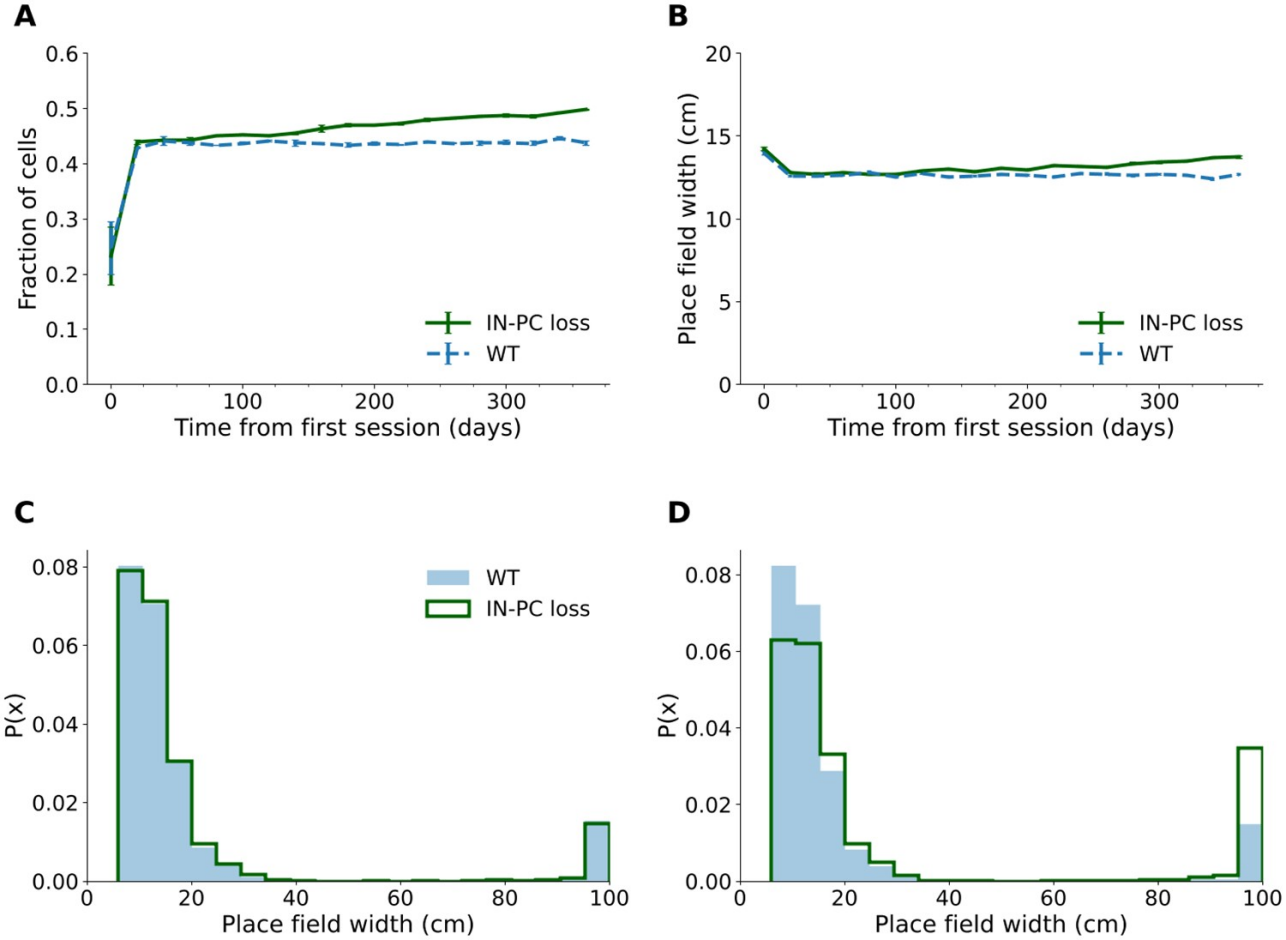
Place cell proportion and mean place field width increased over time when inhibitory synapses were lost. (A) Proportion of place cells over 365 days in the interneuron-to-pyramidal cell (IN-PC) synapse loss and wildtype (WT) model. (B) Mean place field width over 365 days, excluding widths > 50 cm. The means of 2 runs of the simulation are shown in (A) and (B), Error bars show pooled standard error of the mean. Representative probability density functions of place field widths on day 20 (C) and 360 (D).

In our model, this extent of loss of inhibitory synapses caused a gradual increase in the median and interquartile range of mean firing rates over time (day 40: 0.35 ± 0.76 Hz; day 360: 0.58 ± 1.00 Hz), resulting in a significant difference in firing rates to the wildtype (day 360: 0.35 ± 0.76 Hz; *p* < 2.7^−123^, Welch’s two-tailed t-test) (Fig 3C and S2(C) Fig). Furthermore, the fraction of place cells (Fig 5A) and active cells (day 20: 61.1%; day 360: 73.1%), as well as the probability that an active cell remained active between sessions (day 20: 65.4%; day 300: 72.5%) increased gradually. However, there was no difference in the recurrence probability of place cells between sessions 20 days apart (mean over days 20–300: 15.7%). Thus, impaired inhibition increased neuronal activity but did not affect across-session stability.

Although the proportion of place cells in the inhibitory synapse loss model increased over time, the fraction of active cells that form significant place fields decreased from 92.5% to 85.7% between days 20–360. This is likely linked to increased activity, as the proportion of cells with multiple place fields (mean ± SEM days 200–360: wildtype = 3.70% ± 0.04%; IN-PC loss: 4.20%±0.06%, *N* = 18 time points from 2 runs of the simulation per condition), which were not classified as place cells, and cells that were active throughout most of the track (Fig 5D) increased over time.

In addition to more place field formation, the mean place field width slightly increased with time (Fig 5B–5D), indicating a reduced place map resolution as a result of inhibitory synapse loss.

### Excitatory and inhibitory synapse loss impaired place map resolution and stability

When both excitatory and inhibitory synapses were affected, the median neuronal activity also initially increased over time but then started decreasing again (median ± IQR day 40: 0.34 ± 0.75 Hz, day 200: 0.37 ± 0.9 Hz, day 360: 0.35 ± 1.05 Hz) and the range of mean firing rates was higher than in the previous models with either excitatory or inhibitory synapse loss (range of mean firing rates = 0 − 4.94 Hz; GC-PC loss: 0 − 4.76 Hz, IN-PC loss: 0 − 3.79 Hz)(Fig 3D and S2(D) Fig).

**Fig 6:**
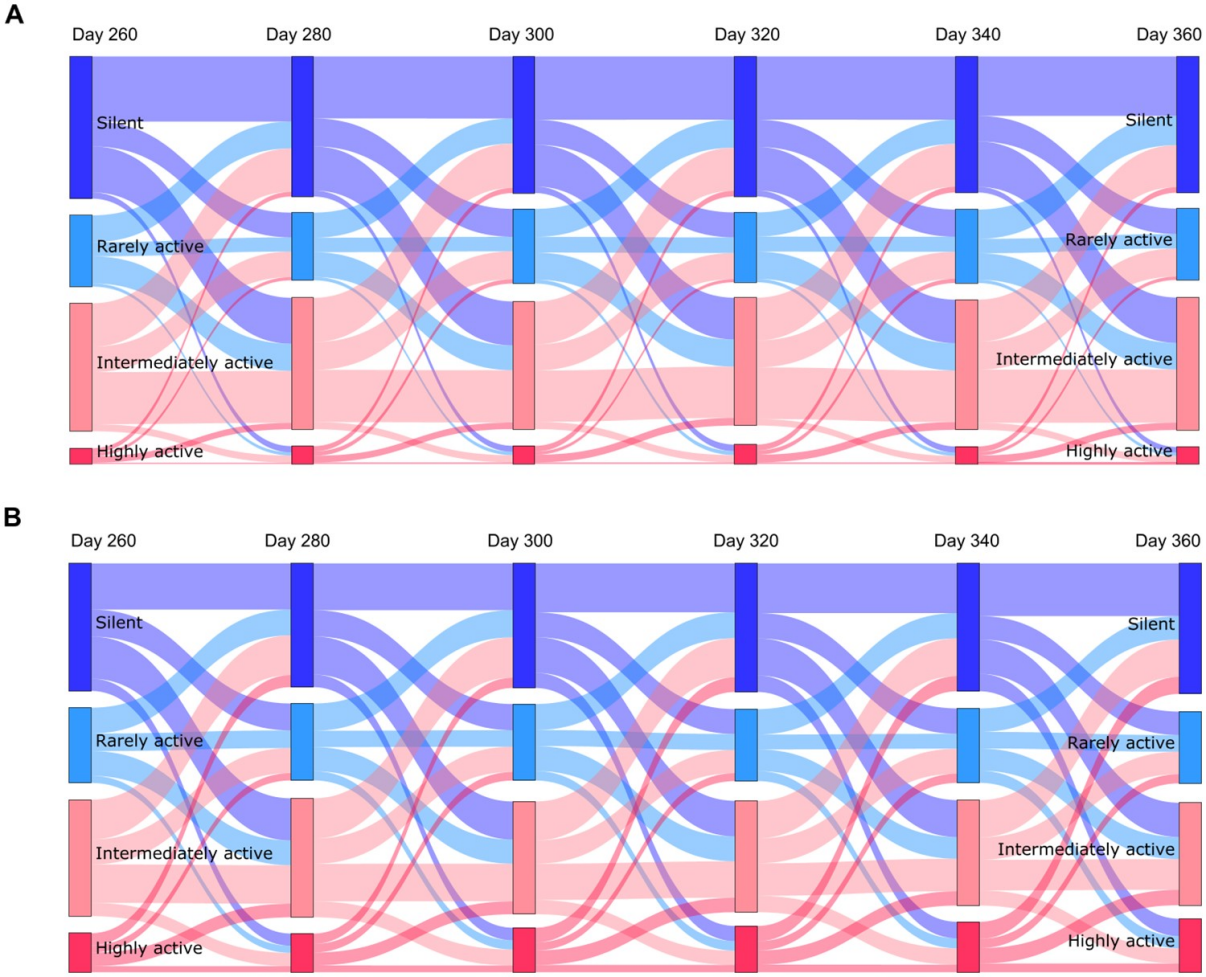
The proportion of highly active cells increases and firing rates are less stable in the synapse loss model. Sankey diagrams showing the proportion of cells classified by mean firing rate and flow between categories across sessions 20 days apart for the wildtype (A) and the synapse loss model (B) starting on day 260. Cells were classified as silent (mean firing rate = 0 Hz; top node in dark blue), rarely active (0 − 0.5 Hz; second node from the top in light blue), intermediately active (0.5 − 2 Hz; second node from the bottom in light pink) and highly active (> 2 Hz; bottom node in red). The height of each node represents the proportion of cells with the given activity level out of all cells on the day specified above the nodes. The height of the links between nodes represents the proportion of cells that were part of the original node on the specified day and were part of the connected node 20 days later. For example, the top left dark blue link represents the proportion of cells that were silent on day 260 and day 280. Links are shaded in the same colour as the node they originate from.

In addition to a progressive increase in the proportion of highly active cells (Fig 3D), more frequent changes in the firing rate could be observed compared to the wildtype model (Fig 6). For instance, in the wildtype just under half of all silent cells remained silent across sessions 20 days apart (as shown by the top dark blue links between nodes in Fig 6A), while this was only true for a third of cells in the excitatory and inhibitory synapse loss model (top dark blue links between nodes in Fig 6B). Nevertheless, activity shifts were mostly gradual in both models, as exemplified by most new highly active cells coming from the pool of ‘intermediately active’ cells (Fig 6).

**Fig 7:**
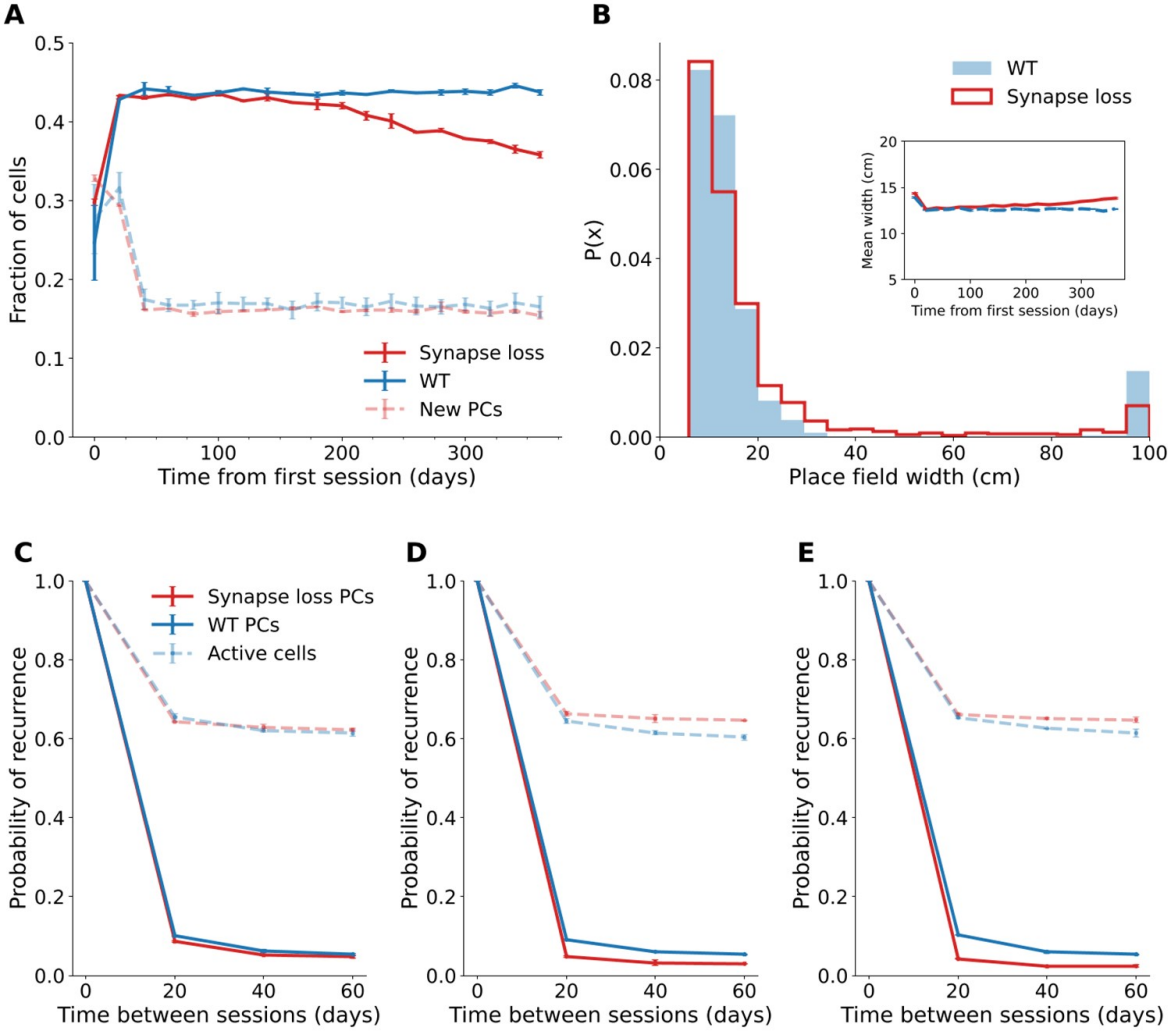
Place cell properties diverge from the wildtype in the double synapse loss model. (A) Proportion of total (dark lines) and new (pale lines) place cells over 365 days in the double synapse loss and wildtype (WT) model. Error bars show standard deviation across two runs of the simulation. (B) Representative probability density function of place field widths on day 360. Inset: Mean field width over 365 days, excluding widths > 50 cm. Error bars show pooled standard error of all place fields across two runs of the simulation. (C-E) Probability of recurrence for active cells and place cells across sessions 20 to 60 days apart from day 20 (C), day 200 (D) and day 300 (E). Error bars show standard deviation across two runs of the simulation.

The proportion of place cells decreased over time in response to excitatory and inhibitory synapse loss, although this increase was smaller than when only excitatory synapses were lost (mean ± SD day 360: = 35.8% ± 0.4%; GC-PC loss day 360: = 30.9% ± 0.7%, means of two runs of the simulation)(Fig 7A). There was a gradual increase in mean place field width (day 20: mean ± SEM = 12.6 ± 0.06 cm, *N* = 6749 place fields; day 360: 13.9 ± 0.07 cm, *N* = 6749 place fields, means of two runs of the simulation)(Fig 7B) comparable to the inhibitory synapse loss model (day 360: 13.73 ± 0.06 cm, *N* = 7754 place fields from two runs of the simulation). Furthermore, there was more variability and abnormal place field sizes than in the wildtype or excitatory or inhibitory synapse loss models (Fig 7B), likely mediated by a lack of inhibition in conjunction with lower excitatory input.

As in the excitatory synapse loss model, the recurrence probability of place cells was gradually reduced (Fig 7C–7E). The density of new place cells remained constant over time (Fig 7A).

## Discussion

In this study, we investigated the effect of AD-related synapse loss on hippocampal network function using a computational model, in which place fields were generated through the interplay between excitatory grid cell input and inhibitory feedback from interneurons. By incorporating homeostatic mechanisms and synaptic connectivity based on experimental findings, the model was able to replicate key aspects of CA1 place cell dynamics. To our knowledge, this is the first model that generates stable place cell dynamics from grid cell inputs long-term. By focusing on place cell function, the model can be used to predict how alterations at the synapse level may affect the emergent properties of the CA1 network.

### Development of the dynamic CA1 network model

To increase place field formation, a local feedback inhibition mechanism and BCM learning were implemented. While Hebbian plasticity is dominantly used in place cell models, the BCM rule has also been shown to support place coding [68, 69]. However, to enable temporal competition using the BCM rule, the rate of synaptic change must be substantially slower than the rate of input presentation, which requires extensive environmental sampling [55]. In contrast, computational modelling suggests fast synaptic changes are required to generate place fields at a realistic timescale [33]. This implies that place coding may involve a learning scheme other than the BCM rule, or multiple learning schemes depending on the input. Alternatively, de Almeida et al. [27] have proposed that place field formation may not rely on synaptic plasticity, except for refinements and long-term stability. They demonstrated place field formation in a grid-cell-to-place-cell model without learning, consistent with close to normal CA1 place coding during exploration in mice with non-functional N-methyl-D-aspartate-receptors (NMDARs), despite exhibiting an NMDAR-dependent form of long-term potentiation [70]. Furthermore, features of BCM learning have been observed in the hippocampus, most notably a dynamic postsynaptic activity-dependent threshold for synaptic strengthening [71, 72]. As such, BCM-like learning remains a plausible alternative to Hebbian learning and has successfully produced the desired grid-cell-to-place-cell transformation at the timescale considered here.

Place cell density was stabilized using a more biologically plausible connectivity, thereby facilitating more realistic feedback inhibition and more dynamic grid cell input through lower synaptic density.

Our model also incorporated synaptic scaling, for which underlying biophysical mechanisms have been identified, including activity-dependent modulation of glutamate receptor expression [73]. While homeostatic mechanisms operate at a slower timescale *in vivo*, modelling studies commonly speed up homeostasis to stabilize Hebbian learning [74–76]. The mechanisms underlying this temporal contradiction between Hebbian plasticity and homeostasis remain unclear, however, proposed explanations include a yet unidentified rapid compensatory process [74].

In mice, it has been reported that 31% of CA1 pyramidal cells are active, while 20% form significant place fields in an environment [50]. While comparisons are difficult due to differences in experimental setup and the possibility of missing silent cells in *in vivo* recordings, our model, with 61.0% active cells and 43.8% place cells, likely involves a higher proportion of place cells than is observed *in vivo*, even compared to rat estimates [77, 78]. As the fraction of active cells and place cells decreased with increasing cell numbers, this may be due to the small network size.

Place map stability, with a recurrence probability of place cells of 32% and 17% for sessions 5 and 30 days apart, respectively, is comparable to experimental values of 25% and 15% [50]. As observed *in vivo*, the recurrence probability was found to only be moderately time dependent.

The mean place field size of 12.6 cm was relatively small, with reported lengths in 1m linear tracks ranging between 20 − 25 cm [50, 79]. Place fields are generally smaller in computational models [28, 80, 81] and it has been argued that grid cell inputs may not be sufficient to produce realistic field sizes without additional input from weakly spatially-modulated cells or recurrent connections among CA3 place cells [82]. In some studies, place field size has been increased by adjusting the grid scale [80, 81]. Thus, there are multiple strategies that may enable more realistic place field sizes, however, such discrepancies in spatial scale are unlikely to significantly affect the network properties [80].

Interestingly, our model generated some non-spatially tuned pyramidal cells, which were active throughout most of the track, as shown in Fig 2D, and not classified as place cells here. Such firing patterns in pyramidal cells are also observed in experimental recordings of place cell activity, but are usually not classified as place fields, for instance by introducing the condition that the spatial information of the cell needs to be higher than when the spatial bins are randomly shuffled [49, 83]. As the occurrence of such non-spatially tuned cells arose after scaling of the network, they may arise as a result of local network interactions due to the connectivity and/or small size of the network presented here. For instance, it may be possible that cells in our network are subject to varying levels of competition within their local network due to the small size of the network, in which case cells with low spatial tuning, whose firing would have been inhibited by a global feedback inhibition loop, escape inhibition due to being in a less competitive local interneuron network. This possibility is supported by the observed increase in non-spatially tuned cells in response to inhibitory synapse loss (Fig 5C and 5D), suggesting that a reduction in the size of the local inhibitory network increases the likelihood of such firing patterns. It would be interesting to determine whether these firing patterns also occur in realistically-sized CA1 networks as a result of local competitive network interactions or whether this simply arises from the small size of our network.

Ziv et al. [50] have found that place cells generally maintain identical place fields *in vivo*, whereas our model exhibited a high level of remapping, prompting strict positional constraints on place fields considered ‘identical’ across sessions. The proportion of new cells within the place cell ensemble increased with cell number (7,788 pyramidal cells: 30.3%, 11,682 pyramidal cells: 37.9%, 15,575 pyramidal cells: 39.3%), indicating the involvement of a larger pool of cells. Thus, the frequent remapping may have been caused by the small network size.

### Excitatory synapse loss may reduce place map stability

Progressive loss of excitatory synapses caused synaptic weight rearrangements consistent with compensatory spine growth and glutamate receptor expression observed *in vivo* and in AD patients [64, 65]. Despite synaptic scaling, the median firing rate decreased and there was more firing variability. The decreased activity may have also impacted interneuron function by lowering the inhibition threshold, thereby providing an additional buffer for the firing rate. Lower mean firing rates in combination with some highly active neurons have also been observed in the CA1 region of 3xTg and APP/PS1 mice, in which this was accompanied by lower entropy, a measure of firing pattern diversity, indicating reduced coding capacity [84, 85].

There was a lower proportion of place cells compared to the wildtype, which has also been observed in 6-months-old APP/PS1 and APP-KI mice [23, 24]. Our model also suggests that excitatory synapse loss could mediate reduced stability of place maps. As reduced stability coincides with spatial memory impairments in APP-KI and 3xTg mice [23, 85], which also exhibit synaptic abnormalities [86, 87] but have normal place field sizes [23, 85], it may be a key mechanism contributing to memory deficits in AD.

### Inhibitory synapse loss may link hyperactivity to reduced map resolution

As interneurons play a key role in the spatiotemporal control of neuronal activity [15, 88], disturbances of which correlate strongly with cognitive deficits [24, 89, 90], the ‘GABAergic hypothesis’, which suggests impaired inhibition may be a critical link between the diverse dysfunctions occurring in AD, has gained popularity in recent years [15].

The increase in firing rate, proportion of highly active cells and cells that were active throughout the whole track is consistent with the hyperactivity observed in CA1 *in vivo* [91]. Place fields were larger and showed increased variability compared to the wildtype, as has been observed in Tg2576 and APP/TTA mice in conjunction with lower spatial information content [22, 25]. Increased place cell number and place field size indicate a lower resolution place map, as hypothesised to occur in experimental models [25].

### Excitatory and inhibitory synapse loss may distinctly contribute to network dysregulation

Korzhova et al. [92] recently identified a progressive increase in activity of intermediately active cells as the primary source of highly active neurons in the cortex of APP/PS1 mice, which was also observed in our model, supporting their hypothesis that network pathology may stem from stable aberrant activity of single cells.

When both excitatory and inhibitory synapses were affected, the resolution and stability of the place map were significantly diminished, accompanied by more variability in neuronal activity between cells. This combination of dysfunctions has also been detected in an APP transgenic model [25], and in a mouse model overexpressing synaptojanin-1 (Synj1), a regulator of synaptic function, which has been implicated in AD [93, 94]. This provides further support for synaptic dysfunction underlying place cell abnormalities and suggests that both excitatory and inhibitory synaptic dysfunction contribute to place cell dysfunction in AD and produce distinct impairments in environmental representation.

### Implications for future work

The link established here between synaptic dysfunction and cognitive impairments, in which synaptic dysfunction was shown to be sufficient to induce place cell abnormalities thought to underlie navigational deficits, is in line with current theories implicating impaired functional connectivity as the major driver of AD pathogenesis [15, 19, 52].

Based on our findings, we predict that hyperactivity in CA1 should coincide with place cell abnormalities and spatial impairments. Furthermore, we hypothesise that impaired spatiotemporal control of pyramidal cell firing by interneurons may be a major contributor to abnormal place field size. Similarly, decay of excitatory EC inputs may underlie reduced ensemble stability. An experimental study employing EC lesions supports our finding that place map stability is reduced in the absence of such inputs, however they also reported increased place field sizes [95]. Recent evidence suggests pyramidal cells in EC layer II also directly innervate CA1 interneurons and that synapses between these populations are selectively lost in Tg2576-APPswe mice [96]. The Tg2576-APPswe model had enlarged place fields, which could be rescued by optogenetic activation of the EC-interneuron synapses. As such, place field enlargement in response to EC lesions may have been mediated by impaired interneuron function.

The relative contribution of excitatory and inhibitory synapse dysfunction in AD is still unclear but may enable the identification of new therapeutic targets. Due to the high inter-connectivity within the hippocampal circuit, addressing this question experimentally requires precise silencing of synaptic transmission, however silencing with the required spatiotemporal control is challenging, potentially limiting the feasibility of such experiments [97].

### Comparison to other place cell models

Most place cell models to date have focused on properties of place cells at shorter timescales, ranging from single sessions to only a few iterations. Such transformation models have been used to investigate properties such as global and rate remapping [32, 98, 99], spike phase precession [100] and memory replay [101]. In contrast, the aim of this investigation was to understand how network properties are maintained in the presence of synaptic restructuring and the effect of AD-like synaptic perturbations. Our model demonstrates how place cell models may be used to interrogate the effect of pathogenic network perturbations on place cell function and spatial memory, a powerful tool given the vast array of published place cell models.

Rennó-Costa and Tort [102] have also examined place cell stability over multiple simulations. In their model, place cells receive 90% of their input from “input cells”, which provide the animal’s position and contextual information to place cells and are meant to represent non-grid inputs from the lateral and medial entorhinal cortex. They also introduced a feedback loop between place cells and grid cells and examined the effect of minor input from grid cells on place cell function in such a context. They found that over 64 simulations, grid cell inputs greatly stabilized place cells in the event of high levels of noise and low input consistency in non-grid inputs. Their findings suggest that grid cells contribute to the robustness of spatial representation, which is in agreement with the experimentally observed increase in place field stability with maturation of the grid cell network in rodents [103]. Their model differs greatly from ours in incorporating only a minor input from grid cells, allowing for place cell feedback to grid cells, and incorporating specific input about position and context through non-grid cell inputs. In the future, it would be interesting to re-examine the effect of grid cells on place cell stability as more information becomes known about the firing pattern and information that non-grid cells provide to place cells.

### Limitations and potential extensions to the model

A biophysical model would enable examination of other interesting network properties observed in AD, such as hypersynchrony [104]. The model could also be further improved by including synaptic weights, synaptic homeostasis and an explicit firing rate for the interneurons, ideally also accounting for the diversity of interneuron types in CA1. Homeostatic inhibitory mechanisms, including altered receptor levels and GABAergic synaptic sprouting have been reported *in vivo* [105, 106]. Furthermore, it would be interesting to extend the model to incorporate place cell remapping, as this has been found to be impaired in AD [23].

A general limitation of grid-cell-to-place-cell feedforward networks is recent evidence for place-cell-to-grid-cell feedback, including the disappearance of grid cell firing in response to place cell inactivation, although the function of this feedback remains unclear [107]. Furthermore, our model assumes that grid cells are the major determinant of place field formation, a view which has been challenged by recent experimental findings, including the formation of place fields after grid cell disruption [95, 108] and the earlier emergence of place cell firing during rat development [109, 110]. Input from subcortical structures, the pre- and parasubiculum and CA3 have all been found to contribute to the spatial tuning of CA1 place fields [111–114]. Spatial input from CA3, for instance, is essential for rapid contextual learning in novel environments, temporal coding at the population level and memory retrieval [45, 115]. In light of this evidence, Bush et al. [111] have proposed that place cell firing is determined by a variety of sensory inputs, primarily boundary cells from the medial entorhinal cortex, with grid cells providing complementary self-motion-derived input to support precise navigation. However, the relative contribution of different cell populations to CA1 place coding remains subject to active research and may even be dynamic, varying with condition or state [116]. Furthermore, Azizi et al. [37] have proposed an alternative explanation, in which grid cell inputs are sufficient to account for place cell firing. They hypothesise that the transformation of grid cell inputs to place cell firing is highly robust to biological noise in the grid cell activity, such that place cell firing is maintained even in the absence of periodicity of grid cells. Using neural network simulations of grid-cell-to-place-cell transformation with various types of noise in the grid cell activity, they demonstrated that place cell firing is indeed robust to such variations, suggesting that given current experimental evidence, it remains plausible that grid cells are the primary source of spatial input to hippocampal place cells. Importantly, grid cells and place cells both show stable firing patterns in familiar environments [47], which are robust to minor changes in the environment [21, 38], while input from other cell types, notably boundary cells in the medial entorhinal cortex, has been observed to be of great importance in novel environments [117]. Thus, grid cell inputs may be especially important in the context of familiar environments, as was investigated here.

While synaptic loss has been found to occur prior to the formation of amyloid plaques [118], plaque proximity-dependent loss has been reported in mouse models [119–121]. As such, it would also be interesting to explore the effect of concentrated areas of synaptic loss. In addition, shrinking cell population sizes, affecting interneurons as early as 6-to-12-months of age, have been detected *in vivo* [67, 122–124], and may contribute to network dysfunction.

Overall, our model shows that synapse loss in CA1 is sufficient to generate the network abnormalities observed in experimental models and AD patients, including hyperactivity [91, 104]. Furthermore, the resulting network abnormalities were shown to induce place cell dysfunction, which has been hypothesised to underlie spatial memory impairment in AD. Thus, our model shows synapse loss is sufficient to drive progressive network dysfunction and impaired spatial representation.

## Methods

We developed a grid cell-to-place cell transformation model based on the model presented by Acker et al. [28]. We termed our implementation of their model consisting of 10,000 grid cells and 2,000 pyramidal cells ‘Model 1’. An expansion of this model with local feedback inhibition and the BCM learning rule is referred to here as ‘Model 2’ and our final model with scaled network connectivity was termed ‘Model 3’.

Each day of the simulation involved the following order of steps: 1. Feedback inhibition, 2. Synaptic plasticity and homeostatic scaling, 3. Feedback inhibition, 4. Synaptic turnover. Where AD-related synaptic loss was implemented, the order from day 1 was as follows: 1. Excitatory synapse loss, 2. Inhibitory synapse loss, 3. Feedback inhibition, 4. Synaptic plasticity and homeostatic scaling, 5. Feedback inhibition, 5. Synaptic turnover.

### Grid cell simulation

We simulated a 1 metre linear track, with cells characterized by their firing rate in each 1 cm bin. Grid cells were simulated as described by Blair et al. [125] (Eq 1), such that their firing rate varied in a hexagonal grid with orientation, phase and offset randomly chosen from a uniform distribution,

$$\begin{array}{c} x_{j}(p,\lambda ,\theta ,c)=g(\sum_{k=1}^{3}\cos(\frac{4\pi }{\lambda \sqrt{3}}u(\theta_{k}+\theta )\cdot (p-c))), \end{array}$$ (1)

where *x*_*j*_(*p*, λ, *θ*, *c*) is the firing rate of grid cell *j* at position *p* in two-dimensional space with inter-vertex spacing λ, angular offset *θ* and spatial phase *c* in two-dimensional space. λ varies between 20 − 100 cm, *c* ranges between 0 − 100 cm in two dimensions and *θ* is randomly chosen to equal 0°, 20° or 40°. ‘·’ indicates the dot product operator. The function *u*(*θ*_*k*_) is given by [cos(*θ*_*k*_), sin(*θ*_*k*_)], such that the cosine function gives a pattern of alternating maxima and minima in direction *θ*_*k*_. The hexagonal grid is generated by the sum of cosine patterns *θ*_1_ = −30°, *θ*_2_ = 30° and *θ*_3_ = 90°. *g*(*x*) = exp[*a*(*x* − *b*)] − 1 is a monotonically increasing gain function with *b* = −3/2 and *a* = 0.3, giving a firing rate of 0 − 3 Hz. Parameters were adapted from de Almeida et al. [27].

### Pyramidal cell simulation

Pyramidal cell activity was modelled as the sum of excitatory grid cell inputs [27] and determined for each 1cm bin in the enclosure.

$$\begin{array}{c} y_{i}(p)=\vec{x}(p)\cdot {\vec{w}}_{i}, \end{array}$$ (2)

where *y*_*i*_(*p*) is the firing rate of pyramidal cell *i* at position *p* in Hz, $\vec{x}\left(p\right)$ is the vector of firing rates of all grid cells that project to pyramidal cell *i*, at position *p*, and ${\vec{w}}_{i}$ is the vector of synaptic weights converging onto pyramidal cell *i*.

### Synaptic weights

The initial weight *w*_*ij*_(*s*) of a synapse between grid cell *j* and pyramidal cell *i* was assumed to vary with synaptic area *s* ranging between 0 − 0.2* μ*m^2^, as described by de Almeida et al. [27],

$$\begin{array}{c} w_{ij}(s)=\frac{s}{0.2}(\frac{s}{s+0.0314}). \end{array}$$ (3)

The pool of synaptic sizes s was determined using the probability density of the synaptic area of excitatory synapses onto granule cells given by [27] in Eq 4. We generated a pool of of 1 million values for s, drawn from a uniform distribution ranging between 0 and 0.2, as well as probability cut-off values for each value of s, drawn from a uniform distribution ranging from 0 to 23. The value of s was added to our synaptic area pool when P(s) was larger than or equal to the assigned probability cut-off.

$$\begin{array}{c} P(s)=A[1-\exp(-\frac{s}{\sigma_{1}})][\exp(-\frac{s}{\sigma_{2}})+B\exp(-\frac{s}{\sigma_{3}})], \end{array}$$ (4)

with *A* = 100.7, *B* = 0.02, *σ*_1_ = 0.022*μ*m^2^, *σ*_2_ = 0.018*μ*m^2^, and *σ*_3_ = 0.15*μ*m^2^, as determined by Trommald and Hulleberg [126].

### Feedback inhibition

The effects of the diverse interneuron populations observed experimentally were approximated via one homogeneous population of ‘typical’ interneurons. Feedback inhibition was based on a model in which the first pyramidal cell to fire within a gamma-oscillation cycle excites an interneuron, which then inhibits the activity of later firing pyramidal neurons. In this rate-based model, it was assumed that neurons with greater excitatory input, and thus higher firing rates, are more likely to quickly overcome the decaying inhibition at the start of each gamma-oscillation cycle, and therefore fire earlier within each cycle [28]. In the mode of inhibition employed in models 1 through 3, referred to as ‘E%-max’ mode, cells were inhibited if their firing rate was not within some fraction of the excitation of the most excited cell [27]. E% was approximated to account for feedback delay, determined by the ratio of the membrane time constant to the time lag between a cell spiking and the incidence of feedback inhibition, which approximately gives E% = 10% [63]. Where specified, we describe the results of implementing a ‘winner-takes-all’ feedback inhibition mode instead, in which only the most excited cell, or cells tied for maximum activity, escape inhibition.

In model 1, a global feedback inhibition mechanism compared pyramidal cell firing rates across the entire track. As specified in the Results section, local feedback inhibition was implemented in models 2 and 3 by incorporating position-specific inhibition within each bin along the track.

Thus, a pyramidal cell was inhibited by setting its firing rate at position p to 0 when the condition

$$\begin{array}{c} y_{i}(p)<(1-k)\max(y^{r}(p)), \end{array}$$ (5)

was satisfied, where *y*_*i*_(*p*) is the the firing rate of pyramidal cell *i* at position *p*, *k* determines the fraction (1 − *k*) of cells that escape inhibition, with *k* = 0 in the ‘winner-takes-all’ case and *k* = 0.1 in the ‘E%-max’ case, and *y*^*r*^(*p*) is the firing rates of all cells that project to interneuron *r*, at position *p*.

### Network architecture

Model 1 and 2 were comprised of 10,000 grid cells and 2,000 pyramidal cells, with each pyramidal cell being innervated by 1,200 randomly determined grid cells, approximating the number of spatially-tuned EC cells that project to a CA1 pyramidal cell, as described by Acker et al [28]. The described feedback inhibition mechanism was mimicked by incorporating equal numbers of interneurons and pyramidal cells, with each interneuron receiving information from all pyramidal cells and projecting to a single randomly selected pyramidal cell each.

Where specified in the Results section, the network architecture was updated in model 3 to fit experimental data on relative cell and synapse numbers in CA1 [56]. The network architecture was scaled to 5,000 grid cells, such that there were 7,788 pyramidal cells and 974 interneurons. Each pyramidal cell was innervated by 31 grid cells and 3 interneurons, with each interneuron being innervated by 19 pyramidal cells. Synaptic connections were determined randomly. For instance, for each pyramidal cell, 3 interneurons were randomly selected out of the entire population of interneurons to innervate the pyramidal cell.

### Synaptic plasticity

Synaptic weights between grid cells and pyramidal cells were updated once a day using either Hebbian learning (model 1) or BCM learning (model 2 and 3). In either case, feedback inhibition immediately preceded learning, such that only the activity of pyramidal cells that escape inhibition is taken into account.

#### Hebbian learning

In model 1, synaptic weights *w*_*ij*_ between grid cell *j* and pyramidal cell *i* were updated once a day using Hebbian learning,

$$\begin{array}{c} \frac{{dw}_{ij}}{dt}=\left(x_{j}\left(\vec{p}\right)\;\cdot \;y_{i}\left(\vec{p}\right)\right)\;\eta , \end{array}$$ (6)

where $x_{j}(\vec{p})$ is the vector of firing rates of grid cell *j* at all positions *p*, $y_{i}\left(\vec{p}\right)$ is the vector of firing rates of place cell *i* at all positions *p*, and *η* = 0.003 is the learning rate [28]. ‘·’ indicates the dot product operator.

#### BCM learning

In model 2 and 3, synaptic weights were instead updated once a day according to the BCM rule [55], As the BCM rule can decrease synaptic weights, any potential negative weights were set to zero immediately after learning to keep all grid cell inputs excitatory.

$$\begin{array}{c} \frac{{dw}_{ij}}{dt}=x_{j}(\vec{p})\;\cdot \;\phi (y_{i}(\vec{p}),\xi ), \end{array}$$ (7)

where $\xi ={\left(\frac{\bar{y_{i}}}{y_{0}}\right)}^{2}\bar{y_{i}}$ is the modification threshold with positive constant *y*_0_ = 50 and $\bar{y_{i}}$ is the mean firing rate of pyramidal cell *i* over all positions along the track. *ϕ*(*y*, *ξ*) = *y*(*y* − *ξ*) with limit *l* = 2, such that

$$\begin{array}{c} \phi (y,\xi )=\{\begin{array}{cc} -l, & \text{if}\;\phi (y,\xi )\leq -l\\ l, & \text{if}\;\phi (y,\xi )\geq l\\ \phi (y,\xi ), & \text{otherwise}. \end{array} \end{array}$$ (8)

### Homeostatic mechanisms

Multiplicative scaling was applied to synaptic weights after each update by dividing by the sum of all weights converging onto each pyramidal cell and multiplying by the expected sum of synaptic strengths [127]. This value corresponded to the expected value of the sum of *n* random draws from the empirical distribution of weights, where *n* is the number of synapses converging onto each cell. The expected value was determined as the average sum of synaptic weights over 10,000 trials.

### Synapse turnover

Synapses were turned over once a day to achieve a mean synapse lifetime *τ* of 10 days [34]. The number of synapses replaced per cell was derived using the exponential decay model

$$\begin{array}{c} N(t)=N_{0}e^{-\frac{t}{\tau }}, \end{array}$$ (9)

where *N*(*t*) and *N*_0_ are the number of synapses on day *t* and 0, respectively, and the number of synapses replaced per cell corresponds to *N*_0_ − *N*(*t*). The intertrial time was taken to be *t* = 1 day. The number of synapses to be turned over on each iteration was determined at the start of the simulation based on synapse numbers on day 0 and kept fixed throughout the simulation. Naive synapses received random weights from the synaptic strength distribution. In model 3, interneuron-to-pyramidal-cell connections were turned over at the same rate by removing the determined number of synaptic connections to pyramidal cells and replacing them with the same number of synaptic connections to randomly determined pyramidal cells on each iteration.

### AD-related synapse loss

Loss of grid-cell-to-pyramidal-cell synapses and interneuron-to-pyramidal-cell synapses were implemented individually and in combination, as specified, to analyze the effects of excitatory and inhibitory synapse loss on place cell function.

Interneuron-to-pyramidal-cell synapse loss was implemented by randomly eliminating a fraction of synapses each day using the following equation, which was obtained by fitting a quadratic regression to the experimental data on axonal loss in the APP/PS1 model determined by Schmid et al [66]. Δ*S*(*T*) refers to the percentage of synapses lost out of the total number of synapses on day 0 at timepoint *T* months from the first running session,

$$\begin{array}{c} \Delta S\left(T\right)=-0.0532T^{2}-2.2179T. \end{array}$$ (10)

One month consisted of 30 days in our simulation, such that $\Delta S(\frac{5}{30})$ gives the percentage of synapses lost by day 5 of the simulation. On each iteration, the percentage of synapses lost up until the previous day were subtracted from the percentage lost by the current day to determine the number of synapses to be eliminated during the current iteration.

There was no interaction between synapse loss and synapse turnover, such that cells whose synaptic connection was eliminated by AD-related synapse loss could form new, naive synaptic connections as part of the synaptic turnover process.

Grid-cell-to-pyramidal-cell synapses were eliminated according to the following equation, which was obtained by fitting a quadratic regression to the experimentally determined pattern of synaptic density reduction in APP/PS1 mice [64].

$$\begin{array}{c} \Delta S\left(T\right)=-0.2716T^{2}-9.0677T \end{array}$$ (11)

for *T* ≤ 12 months.

### Place field analysis

A significant place field was defined as a single continuous region of 5 − 50 cm (see Results section), in which the cell’s firing rate was within 20% of its maximum firing rate during the running session [28]. For calculating the probability of recurrence of a place cell, a place field was deemed to have been maintained across sessions if the location of the centroid was within 10 cm of the centroid in the previous session if samples were taken every 20 days, or 5 cm if samples were taken every 5 days.

Cells were deemed ‘active cells’ if they had a non-zero firing rate at any position throughout the track.

## Supporting information

S1 Text Additional unsuccessful approaches to stabilize place field density. Describes other approaches aimed to stabilize place cell density including the use of subtractive normalization, incorporation of multiple enclosures and usage of diverse Hebbian learning rates in model 1.
(PDF)

S1 Fig Behaviour of model 3 at higher and lower synaptic turnover rates. (A) Probability of recurrence for place cells and active cells across sessions 20 to 60 days apart from day 20 for model 3 with turnover rate used here (blue), doubled turnover rate (light blue) and half the turnover rate (purple). (B) Proportion of total and new place cells over 120 days. (C) Mean place field width over 120 days, excluding widths > 50 cm. The results of one run of the simulation are shown for each condition.
(TIF)

S2 Fig Histograms of mean firing rate distribution of cells in the wildtype and synapse loss models. Histograms of mean firing rate of cells on day 360 in the wildtype (A), the excitatory synapse loss model (B), the inhibitory synapse loss model (C) and the excitatory and inhibitory synapse loss model (D). The result of a single run of the simulation is shown for each condition.
(TIF)
