# Supplementary material for: A computational grid-to-place-cell transformation model indicates a synaptic driver of place cell impairment in early-stage Alzheimer’s Disease
Source: PLoS Comput Biol. 2021 Jun 16;17(6):e1009115. doi: 10.1371/journal.pcbi.1009115 (PMC8238223; doi:10.1371/journal.pcbi.1009115)
Supplement: S1 Text — Describes other approaches aimed to stabilize place cell density including the use of subtractive normalization, incorporation of multiple enclosures and usage of diverse Hebbian learning rates in model 1. (PDF) [file pcbi.1009115.s001.pdf]

S1 Text.
Additional unsuccessful approaches to stabilize place cell density

Subtractive normalization

As most forms of synaptic homeostasis observed experimentally operate over hours or days [1, 2], the rate of homeostasis was reduced. Multiplicative synaptic scaling does not allow for the homeostasis rate to be adjusted, so subtractive normalization was implemented. Under subtractive normalization, all synapses converging onto a neuron are changed by the same magnitude to keep the total synaptic input fixed, rather than weight adjustments being proportional to individual strength [3], thereby generating more competition [4]. Here, the sum of weights converging onto each cell rarely stabilized at the homeostatic point, as this would have required negative weights, yet the subtractive decay term was not sufficient to effectively limit growth of relatively stronger weights. Thus, subtractive normalization did not elicit the desired weight stabilization.

Subtractive normalization was applied instead of multiplicative scaling after Hebbian learning [5]

$$w_{ij} = w_{ij} - \eta_{homeo} \left(\sum_j w_{ij} - \theta_{homeo} \right), \tag{1}$$

where $\theta_{homeo} = 50$ and η_{homeo} is the homeostatic learning rate, set to 0.1 of the Hebbian learning rate to mimic the relative temporal timescale of Hebbian plasticity and homeostatic mechanisms in the hippocampus [6].

Incorporation of multiple enclosures

Incorporation of multiple enclosures was motivated by theories that place fields from different enclosures may 'mix' in familiar environments [7]. Running sessions in novel environments, represented by distinct grid cell arrangements [8], induced new place fields that were retained in the familiar environment through their effects on synaptic weights. However, continuous introduction of new enclosures was required to prevent stabilization at a specific subset of place cells, but still resulted in a general decline with occasional spikes in place cell number. These spikes were likely mediated by close correspondence of the novel and familiar grid inputs, facilitating rapid place field formation.

Diverse synaptic plasticities with Hebbian learning

It has also been proposed that the co-existence of more and less stable stimulus responses may result from a diversity in synaptic plasticity [9]. Indeed, applying diverse learning rates across cells resulted in more place field formation than the BCM rule alone, however, again, it was not sufficiently high for stabilization. Furthermore, there was a stronger afferent synaptic strength divide, such that cells with slow plasticity were generally outcompeted.

References

1. Turrigiano GG. The dialectic of hebb and homeostasis. Philosophical Transactions of the Royal Society B: Biological Sciences. 2017;doi:10.1098/rstb.2016.0258.

2. Watt AJ, Desai NS. Homeostatic plasticity and STDP: Keeping a neuron's cool in a fluctuating world. Frontiers in Synaptic Neuroscience. 2010;doi:10.3389/fnsyn.2010.00005.

3. Elliott T. An analysis of synaptic normalization in a general class of Hebbian models. Neural Computation. 2003;doi:10.1162/08997660360581967.

4. Abbott LF, Nelson SB. Synaptic plasticity: Taming the beast. Nature Neuroscience. 2000;doi:10.1038/81453.

5. Miller KD, MacKay DJC. The Role of Constraints in Hebbian Learning. Neural Computation. 1994;doi:10.1162/neco.1994.6.1.100.

6. Zenke F, Gerstner W, Ganguli S. The temporal paradox of Hebbian learning and homeostatic plasticity. Current Opinion in Neurobiology. 2017;doi:10.1016/j.conb.2017.03.015.

7. Agmon H, Burak Y. A theory of joint attractor dynamics in the hippocampus and the entorhinal cortex accounts for artificial remapping and grid cell field-to-field variability. eLife. 2020;doi:10.7554/elife.56894.

8. Fyhn M, Hafting T, Treves A, Moser MB, Moser EI. Hippocampal remapping and grid realignment in entorhinal cortex. Nature. 2007;doi:10.1038/nature05601.

9. Sweeney Y, Clopath C. Population coupling predicts the plasticity of stimulus responses in cortical circuits. eLife. 2020;doi:10.7554/eLife.56053.
