# Supplementary figures and images for: A computational grid-to-place-cell transformation model indicates a synaptic driver of place cell impairment in early-stage Alzheimer’s Disease
Source: PLoS Comput Biol. 2021 Jun 16;17(6):e1009115. doi: 10.1371/journal.pcbi.1009115 (PMC8238223; doi:10.1371/journal.pcbi.1009115)

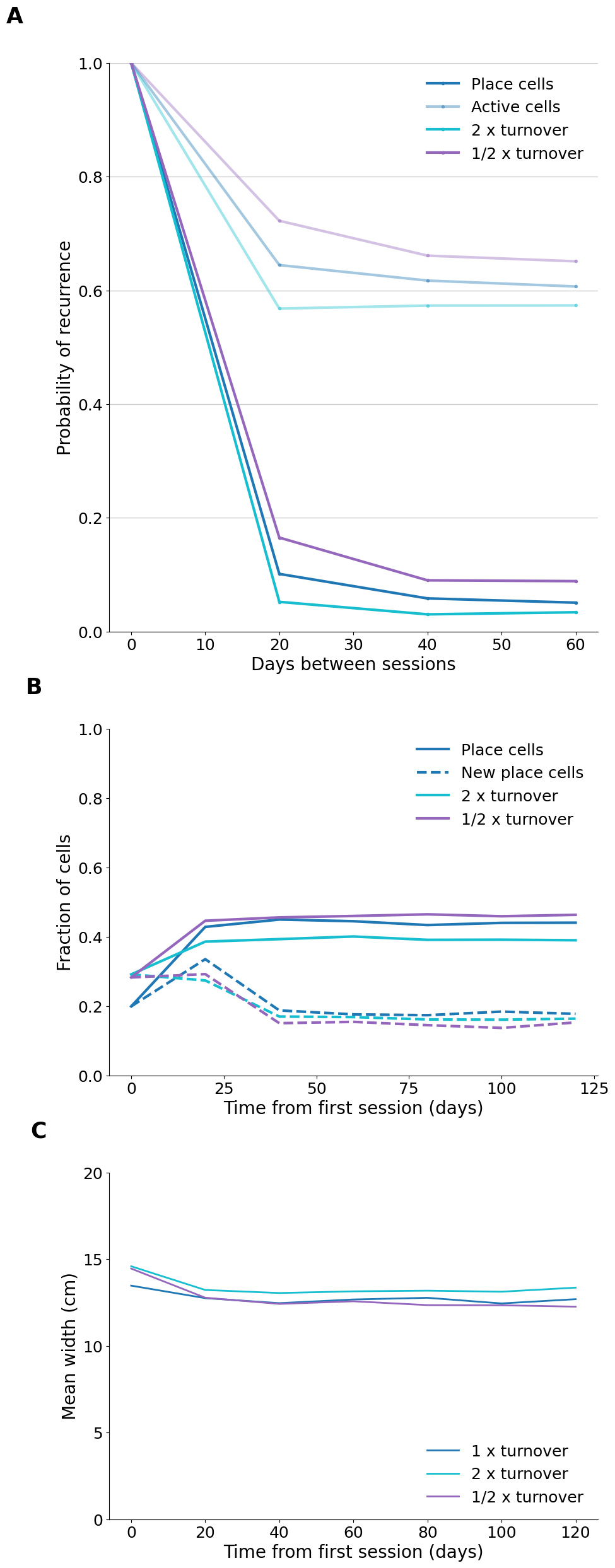

Supplement: S1 Fig — (A) Probability of recurrence for place cells and active cells across sessions 20 to 60 days apart from day 20 for model 3 with turnover rate used here (blue), doubled turnover rate (light blue) and half the turnover rate (purple). (B) Proportion of total and new place cells over 120 days. (C) Mean place field width over 120 days, excluding widths > 50 cm. The results of one run of the simulation are shown for each condition. (TIF) [file pcbi.1009115.s002.tif]

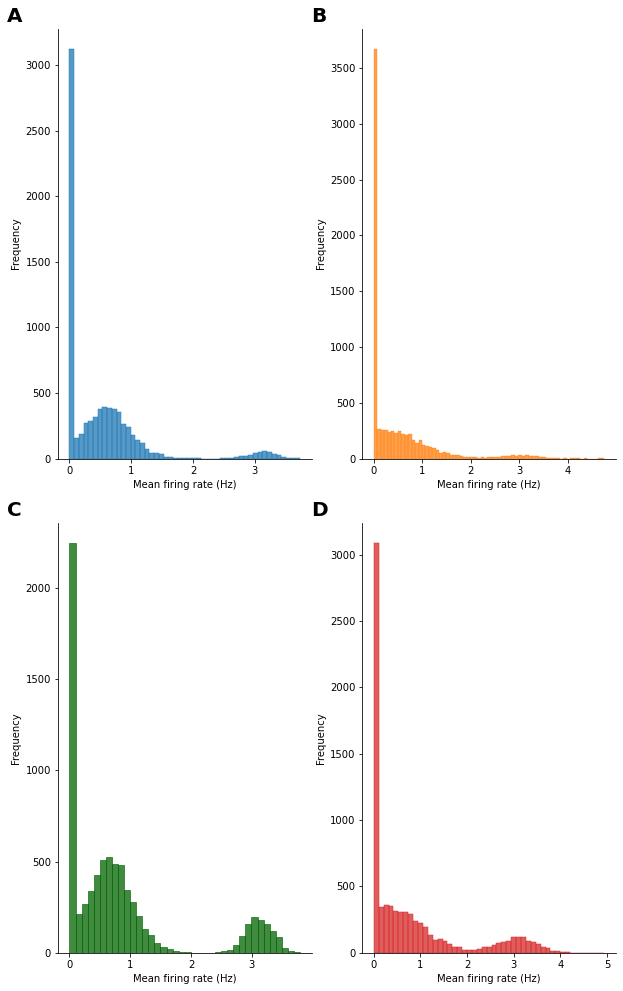

Supplement: S2 Fig — Histograms of mean firing rate of cells on day 360 in the wildtype (A), the excitatory synapse loss model (B), the inhibitory synapse loss model (C) and the excitatory and inhibitory synapse loss model (D). The result of a single run of the simulation is shown for each condition. (TIF) [file pcbi.1009115.s003.tif]
